# Supplementary material for: Modeling glioblastoma heterogeneity as a dynamic network of cell states
Source: Mol Syst Biol. 2021 Sep 16;17(9):e10105. doi: 10.15252/msb.202010105 (PMC8444284; doi:10.15252/msb.202010105)
Supplement: Supplementary file 6 — Source Data for Figure 5 [file MSB-17-e10105-s004.zip › Figure5A_sourcedata/GSEA_3071/hallmarks_stateII.GseaPreranked.1623742536692/HALLMARK_HEDGEHOG_SIGNALING.html]

Details for gene set HALLMARK\_HEDGEHOG\_SIGNALING[GSEA]

|  || Dataset | state23071 |
| Phenotype | NoPhenotypeAvailable |
| Upregulated in class | na\_neg |
| GeneSet | HALLMARK\_HEDGEHOG\_SIGNALING |
| Enrichment Score (ES) | -0.47978494 |
| Normalized Enrichment Score (NES) | -1.7320168 |
| Nominal p-value | 0.034615386 |
| FDR q-value | 0.11253377 |
| FWER p-Value | 0.243 |
Table: GSEA Results Summary

  

Fig 1: Enrichment plot: HALLMARK\_HEDGEHOG\_SIGNALING      
 Profile of the Running ES Score & Positions of GeneSet Members on the Rank Ordered List

  

| PROBE | GENE SYMBOL | GENE\_TITLE | RANK IN GENE LIST | RANK METRIC SCORE | RUNNING ES | CORE ENRICHMENT || 1 | TLE3 |  |  | 215 | 0.861 | 0.0093 | No |
| 2 | MYH9 |  |  | 323 | 0.735 | 0.0361 | No |
| 3 | GLI1 |  |  | 464 | 0.644 | 0.0481 | No |
| 4 | ETS2 |  |  | 923 | 0.466 | -0.0306 | No |
| 5 | NRP1 |  |  | 1372 | 0.362 | -0.1144 | No |
| 6 | L1CAM |  |  | 1402 | 0.357 | -0.0958 | No |
| 7 | ADGRG1 |  |  | 2239 | -0.252 | -0.2825 | No |
| 8 | HEY2 |  |  | 2505 | -0.286 | -0.3268 | No |
| 9 | HEY1 |  |  | 3130 | -0.408 | -0.4504 | Yes |
| 10 | CNTFR |  |  | 3176 | -0.422 | -0.4311 | Yes |
| 11 | TLE1 |  |  | 3225 | -0.434 | -0.4115 | Yes |
| 12 | RTN1 |  |  | 3232 | -0.436 | -0.3816 | Yes |
| 13 | CDK6 |  |  | 3466 | -0.526 | -0.4008 | Yes |
| 14 | VEGFA |  |  | 3532 | -0.560 | -0.3764 | Yes |
| 15 | CRMP1 |  |  | 3583 | -0.594 | -0.3459 | Yes |
| 16 | DPYSL2 |  |  | 3645 | -0.642 | -0.3146 | Yes |
| 17 | SCG2 |  |  | 3975 | -1.137 | -0.3134 | Yes |
| 18 | NRCAM |  |  | 4076 | -2.179 | -0.1809 | Yes |
| 19 | NRP2 |  |  | 4087 | -2.583 | 0.0027 | Yes |
Table: GSEA details [plain text format]

  

Fig 2: HALLMARK\_HEDGEHOG\_SIGNALING: Random ES distribution      
 Gene set null distribution of ES for **HALLMARK\_HEDGEHOG\_SIGNALING**
